# Adequacy evaluation of 22‐gauge needle endoscopic ultrasound‐guided tissue acquisition samples and glass slides preparation for successful comprehensive genomic profiling testing: A single institute experience

**DOI:** 10.1002/deo2.70104

**Published:** 2025-05-09

**Authors:** Tami Nagatani, Yoji Wani, Masahiro Takatani, Soichiro Fushimi, Hirofumi Inoue, Shinichiro Hori, Kyohei Kai, Hideki Yamamoto, Tetsuya Okazaki, Maki Tanioka, Hiroyuki Okada, Akira Hirasawa

**Affiliations:** ^1^ Department of Pathology Japanese Red Cross Society Himeji Red Cross Hospital Hyogo Japan; ^2^ Department of Genetic Medicine Japanese Red Cross Society Himeji Red Cross Hospital Hyogo Japan; ^3^ Clinical Genomic Medicine Dentistry and Pharmaceutical Science Okayama University Graduate School of Medicine, Dentistry and Pharmaceutical Sciences Okayama Japan; ^4^ Department of Internal Medicine Japanese Red Cross Society Himeji Red Cross Hospital Hyogo Japan; ^5^ Division of Medical Support Dentistry and Pharmaceutical Science Okayama University Graduate School of Medicine Okayama Japan

**Keywords:** biliary tract cancer, comprehensive genomic profiling, endoscopic ultrasound‐guided fine needle aspiration, endoscopic ultrasound‐guided fine needle biopsy, pancreatic cancer

## Abstract

**Objectives:**

This study aimed to evaluate the successful sequencing rate of Foundation One CDx (F1CDx) using small tissue samples obtained with a 22‐gauge needle (22G) through endoscopic ultrasound‐guided fine needle acquisition (EUS‐TA) and to propose guidelines for tissue quantity evaluation criteria and proper slide preparation in clinical practice.

**Methods:**

Between June 2019 and April 2024, 119 samples of 22G EUS‐TA collected for F1CDx testing at Himeji Red Cross Hospital were retrospectively reviewed. Tissue adequacy was only assessed based on tumor cell percentage (≥20%). The procedure stopped when white tissue fragments reached 20 mm during macroscopic on‐site evaluation. The specimens were prepared using both ‘tissue preserving sectioning’ to retain tissue within formalin‐fixed paraffin‐embedded blocks and the ‘thin sectioning matched needle gauge and tissue length’ method with calculation to ensure minimal unstained slides for the 1 mm^3^ sample volume criterion. Tissue area from HE slides and sample volume were measured, and F1CDx reports were analyzed.

**Results:**

Of 119 samples, 108 (90.8%) were suitable for F1CDx. Excluding the cases not submitted for testing, in the 45 cases where F1CDx was done using 22G EUS‐TA samples, eight (17.8%) had a sum of tissue area tissue of 25 mm^2^ or greater in the HE‐stained sample. However, all cases met the F1CDx 1 mm^3^ volume criterion by submitting > 30 unstained slides per sample. As a result, 43 of 45 cases (95.6%) were successfully analyzable.

**Conclusions:**

The 22G EUS‐TA needle is an effective tool for providing the sufficient tissue volume required for F1CDx.

## INTRODUCTION

For intra‐abdominal tumors such as pancreatic and liver tumors, histopathological diagnosis is often achieved through tissue sampling using endoscopic ultrasound‐guided tissue acquisition (EUS‐TA), which is highly accurate and reliable.^1^, ^2^ Many cases of pancreatic and biliary tract cancer are inoperable at the time of diagnosis; thus, it is crucial to obtain sufficient tissue for subsequent auxiliary tests such as comprehensive genomic profiling (CGP) testing. EUS‐TA can serve as an important procedure.^3^, ^4^

Foundation One CDx (F1CDx; Foundation Medicine Inc.; FMI) and the OncoGuide NCC Oncopanel system (NCCOP; Sysmex Corporation) are CGP tests that　have been covered by insurance in Japan since June 2019. GenMineTOP (Konica Minolta Realm Inc., Tokyo, Japan) is also a CGP test that has been covered by insurance in Japan since August 2023. These tests differ in the number of genes analyzed and the amount of tissue required. Specifically, F1CDx analyzes 324 genes and NCCOP analyzes 124 genes.

For F1CDx, the required tissue must meet the following criteria: the tissue should have an optimal percentage of nucleated tumor cells of 30% or more and a minimum percentage of 20% or more. Additionally, the tissue should have a surface area of at least 25 mm^2^ or a total volume of at least 1 mm^3^, which corresponds to 10 unstained slide glasses (USS) with a thickness of 0.005 mm.^5^, ^6^ Meanwhile, NCCOP requires that tumor cells comprise more than 20% of the tissue area, which should be more than 4 mm^2^. In addition, the sample must have five USS, with a thickness of 0.01 mm.^7^ Studies are underway to determine whether different EUS‐TA puncture methods can obtain sufficient specimens for CGP.^8^, ^9^, ^10^ The success rate of CGP varies depending on the gauge of the needle used and the area of the obtained tissue fragments.^11^, ^12^, ^13^ However, the unacceptable cases in the pre‐analytic evaluation were numerous, as many cases often did not meet the criteria for tissue area, rather than the tumor cell proportion^13^, ^14^ A recent comprehensive study on the success rate of CGP with F1CDx and NCCOP in routine clinical practice on pancreatic EUS‐TA samples reports that 63 of 109 EUS‐TA cases (57.8%) were histopathologically adequate.^15^ The lower adequacy of the 22G tissue than that of the 19G causes the overall success rate of CGP tests to be lower. Correspondingly, this is also true for biliary tract cancer cases.^16^ Moreover, in both reports, more NCCOP tests were submitted than F1CDx tests, which suggests that the choice of CGP is limited by the size of the needle used. Currently, using 19G needles is recommended for CGP testing, but 22G needles are preferred even for F1CDx because they are easier to puncture compared to 19G needles, and are more widely used.^17^ Only one report focusing on samples obtained with 22G EUS‐TA demonstrated a high success rate of 76.0% (38/50) for CGP; however, only two of 50 (4.0%) samples underwent F1CDx testing.^18^

Until now, there has been no detailed examination of the histopathological adequacy of tissue or the prepared slide specimens themselves. The purpose of this study was to retrospectively review CGP testing of EUS‐TA specimens at our institution and to propose guidelines for histopathological evaluation, and adequate slide preparation that would lead to a high success rate of F1CDx testing analysis in 22G‐obtained tissues.

## METHODS

### Case selection

**FIGURE 1:**
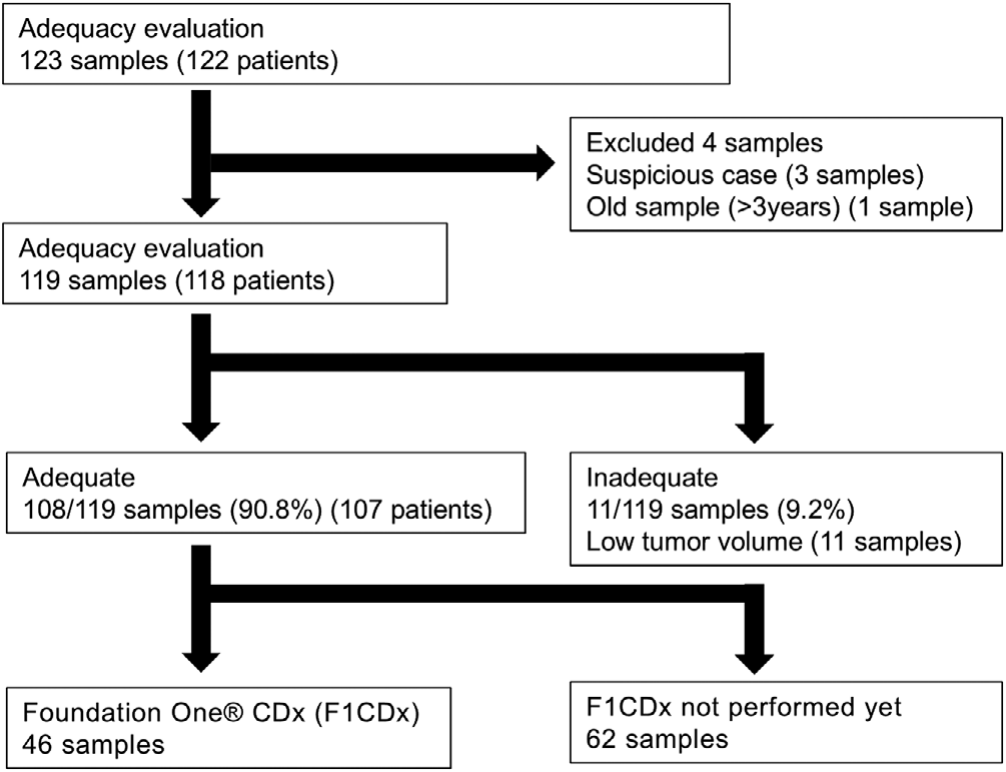
Flow diagram of the study.

Of 559 samples (512 patients) collected with EUS‐TA between June 2019 and April 2024 using the Himeji Red Cross Hospital (HRCH) Pathology Reporting System, 123 samples (122 patients) with a pre‐analytical slide review request for F1CDx examination were included (Figure 1). Information about patients and EUS‐TA procedures was obtained from patient records and the endoscopy system.

### EUS‐TA procedure and specimen processing for pathological diagnosis

**FIGURE 2:**
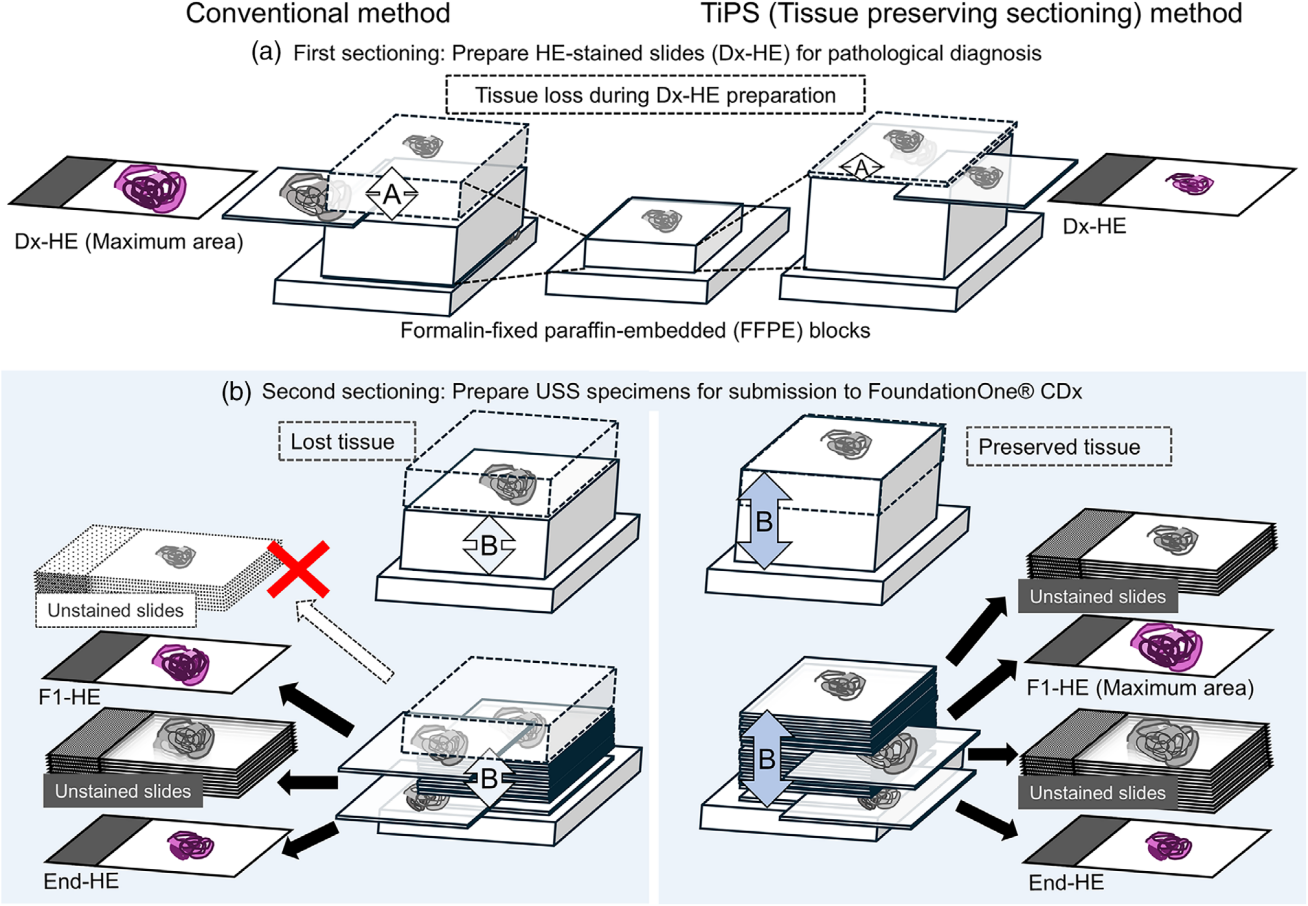
Comparison of Specimen Preparation Between the TiPS (Tissue Preserving Sectioning) Method and the Conventional Method. Dx‐HE, hematoxylin and eosin (HE)‐stained slides for routine pathological diagnosis; F1‐HE, HE‐stained slide prepared by the second sectioning of tissue; End‐HE, HE‐stained slide prepared at the end of the microsectioning process. During sectioning of the formalin‐fixed paraffin‐embedded (FFPE) blocks, Dx‐HE was initially prepared with shallow microsectioning, with the intention that most of the tissue in paraffin remains unexposed. As a result, tissue loss is minimized. This allows for the preparation of a larger number of unstained specimens from the remaining tissue in the FFPE block for subsequent comprehensive genomic profiling testing. In the TiPS method, the FFPE tissue between Dx‐HE and F1‐HE (maximum area) which is lost in the conventional method can be preserved.

In EUS‐TA, basically, a 22‐gauge needle was advanced into the target lesion under EUS guidance, and 20 to‐and‐fro movements were performed within the target lesion. The suction technique with 20 cc of negative pressure was applied. In cases with significant blood reflux, the negative pressure was reduced accordingly. The physician‐dispensed the collected tissue into a Petri dish. Technicians macroscopically observed the tissue using the FUJICOLOR LED Viewer Pro 4 × 5 (FUJIFILM Corporation) to transmit light from below. The examination was stopped when the total length of the whitish macroscopic visible core (MVC) reached at least 20 mm in the macroscopic on‐site evaluation (MOSE) previously described.^19^ The collected tissue fragments were immediately fixed in 10% neutral buffered formalin. After fixation, technicians separated the tissue into white and non‐white portions as much as possible, followed by preparing formalin‐fixed paraffin‐embedded (FFPE) blocks. Conventionally, when preparing HE slides (Dx‐HE) for routine pathological diagnosis, the tissue within the FFPE block is sectioned until most of it is exposed(maximum area). The tissue that is exposed before this point is discarded and is not used for genetic testing. In contrast, in the Tissue Preserving Sectioning (TiPS) method we named and used, Dx‐HE was prepared with the tissue slightly exposed in the FFPE block, leaving intact the majority of the tissue within it and allowing for the preparation of multiple USS for subsequent CGP analysis (Figure 2).

### Pathologists’ pre‐analytical review and adequacy evaluation

In‐house pathologists (Yoji Wani and Soichiro Fushimi) assessed the percentage of tumor cells on Dx‐HE. When the percentage of tumor cells was 20% or more, the sample was considered adequate. Only the percentage of tumor cells was used for evaluation, and area assessment was not performed. If the low tumor cell content might affect the analysis, comments were added regarding the possibility of insufficient DNA content.

### Glass slides preparation for F1CDx

**FIGURE 3:**
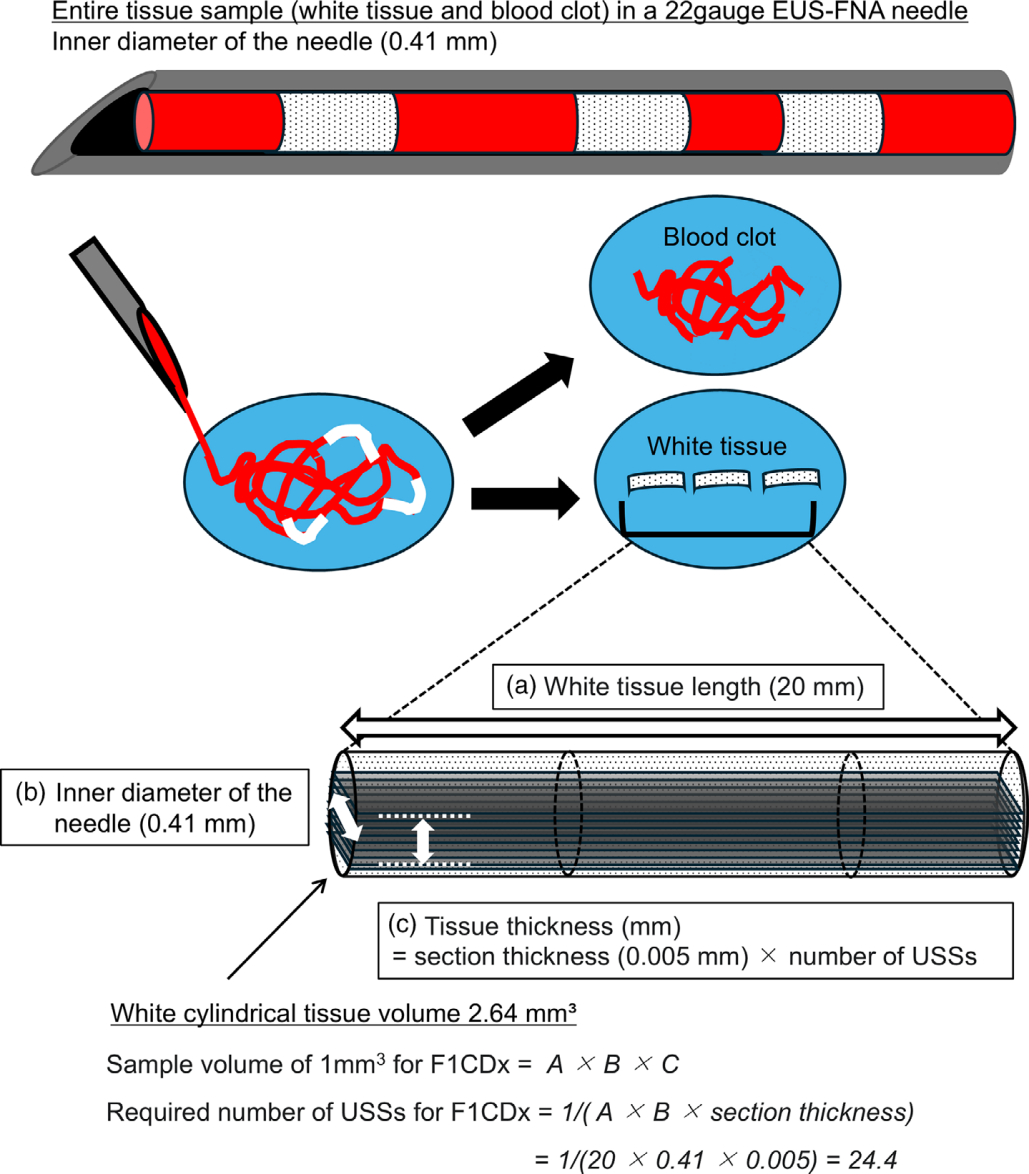
Conceptual diagram of thin sectioning matched needle gauge and tissue length (SMAGLE) and method for evaluating the required number of unstained slide specimens (USS) for Foundation One CDx (F1CDx).

From the remaining FFPE tissue, a second sectioning was performed to prepare a 5‐µm HE‐stained slide (F1‐HE) and more than 10 USS according to F1CDx guidelines.^5^, ^6^ The required number of USS was theoretically calculated (Figure 3). We called this original method “thin sectioning matched needle gauge and tissue length” (SMAGLE) which calculates the minimum required USS based on the long diameter of the needle used and the length of the tissue sample. Briefly, we aimed to prepare a minimum of 25 USS [>1 mm^3^/ (tissue length, 20 mm) x (22G inner diameter, 0.41 mm) x (section thickness, 0.005 mm)]. Some additional USS were prepared in samples on the basis of pathologist comments. One HE‐stained slide was prepared at the end of the microsectioning process (End‐HE).

### Method for measuring tissue area from HE‐stained slides and calculating sample volume

**FIGURE 4:**
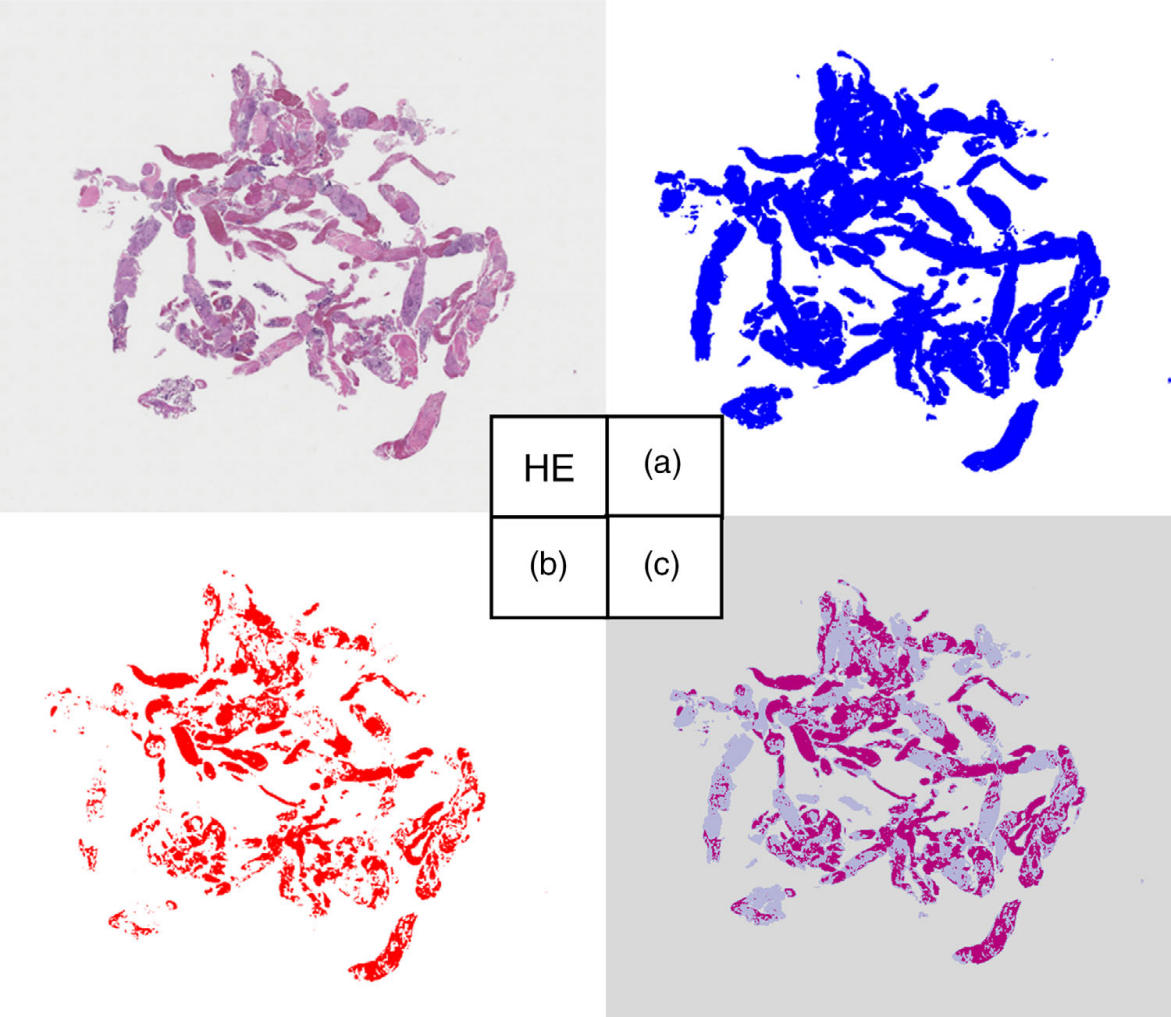
Method for measuring tissue area obtained from Hematoxylin and Eosin (HE) slides. HE, Scanned HE‐stained specimen; (a) All scanned areas are displayed in blue; (b) Blood components are displayed in red; (c) Overlay of a and b. The tissue area was calculated by subtracting b from a.

The tissue area on HE‐stained slides was evaluated as follows. Using the All‐in‐One Fluorescence Microscope BZ‐X800 (Keyence), we scanned the tissue area on the HE‐stained specimen (Figure 4). We combined some scanned images to create a single composite image. We used the quantitative Hybrid Cell Count function to measure tissue area. After identifying the entire tissue using luminance, we colored it blue (Figure 4a: total area). Next, we identified the blood area using a hue, which was colored red (Figure 4b: blood area). We overlaid A and B. We calculated the tissue area in the specimen by subtracting B from A (Figure 4c).

Sample volume (mm^3^) was calculated by multiplying tissue area on F1‐HE by the number of USS and section thickness (0.005 mm; Figure 2).

### Analysis of F1CDx reports

From the F1CDx reports, the tumor ratios, which the FMI pathologists evaluated before performing F1CDx tests, “Pass“ or ”Qualified” as well as the microsatellite status (MSS), tumor mutation burden (TMB), and pathogenic gene mutations identified. Both “Pass” and “Qualified” were considered successful.

### Statistical analysis

Continuous variables are expressed as medians (range). The Kruskal–Wallis test was used for continuous variables (the Holm correction was used in adjustment for multiple comparisons testing among the three groups). Statistical significance was set at *p* < 0.05. Statistical analysis was performed using EZR, version 1.68 (Saitama Medical Center, Jichi Medical University), which is a graphical user interface for R (The R Foundation for Statistical Computing). More precisely, it is a modified version of the R commander designed to add statistical functions frequently used in biostatistics.^20^

## RESULTS

Histopathological evaluation requests were received for 123 samples from 122 patients. Of these, 108 samples (90.8%) were suitable for examination on the basis of the pathologist's pre‐analytical review; 11 samples (9.2%) were considered unsuitable due to low tumor cell percentage (Figure 1).

**FIGURE 5:**
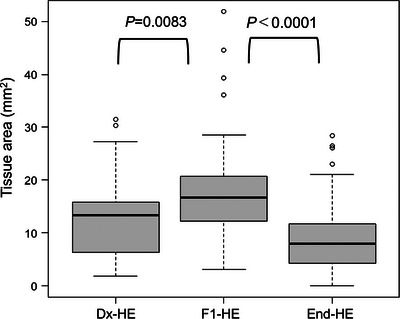
Comparison of tissue area among Hematoxylin and eosin (HE)‐stained slides for routine pathological diagnosis (Dx‐HE), HE‐stained slide prepared by the second sectioning of tissue (F1‐HE), and HE‐stained slide prepared at the end of the microsectioning process (End‐HE). The *p*‐values for tissue area comparisons between the Dx‐HE and F1‐HE groups (0.0083), and between the F1‐HE and End‐HE groups (<0.0001), show that the tissue area in the F1‐HE group was significantly larger.

In total, 46 samples from 45 patients were submitted for F1CDx (Table 1). The patients ranged in age from 43 to 82 years, with an average age of 64 years. The male‐to‐female ratio was 27:19. The submitted samples included 38 samples of pancreatic ductal adenocarcinoma (PDAC), one sample of pancreatic neuroendocrine carcinoma (PNEC), and seven samples of biliary tract adenocarcinoma (BTAC). One was a discontinued patient. Among the 45 samples, 27 had tumor diameter ≤30 mm, which included 25 pancreatic samples (head, 10; body, 14; tail, 11), five lymph node samples, one liver sample, two biliary tract samples, and two other samples (Table 2). All puncture needles were 22G. The most used needle type was Acquire (Boston Scientific). Regarding the number of needle punctures, there were two or fewer for 26 patients, three for 10 patients, and four or more for nine patients (mean, 2.6 punctures). Eleven samples were adequate but included comments indicating possible insufficient DNA quantity. The *p*‐values for tissue area comparisons between the Dx‐HE and F1‐HE groups (0.0083), and between the F1‐HE and End‐HE groups (<0.0001), show that the tissue area in the F1‐HE group was significantly larger (Figure 5). In eight of 45 samples (17.8%), the F1‐HE area exceeded the 25 mm^2^ standard required by F1CDx (Table 3).

**TABLE 1:** Clinical summary (*n* = 46).

	Total *n* = 123	F1CDx cases *n* = 46
Age, years median (range)	69 (31–85)	63 (43–82)
Sex		
Male, *n* (%)	67^*^ (54.5)	27^*^ (58.7)
Female, *n* (%)	56 (45.5)	19 (41.3)
Histological type		
Pancreatic epithelial neoplasms		
Invasive ductal carcinomas		
Adenocarcinoma (PDAC), *n* (%)	97^*^ (78.9)	38^*^ (82.6)
Adenosquamous carcinoma, *n* (%)	2 (1.6)	0 (0)
Mucinous carcinoma, *n* (%)	0 (0)	0 (0)
Anaplastic carcinoma, *n* (%)	0 (0)	0 (0)
Acinar cell neoplasms		
Acinar cell carcinoma, *n* (%)	1 (0.8)	0 (0)
Neuroendocrine neoplasms		
Neuroendocrine tumors (PNEC), *n* (%)	1 (0.8)	0 (0)
Neuroendocrine carcinoma, *n* (%)	1 (2.2)	1 (2.2)
Mixed neoplasm		
Mixed neuroendocrine non‐neuroendocrine neoplasms, (G3), *n* (%)	1 (0.8)	0 (0)
Mixed ductal‐neuroendocrine carcinoma	1 (0.8)	0 (0)
Biliary tract adenocarcinoma (BTAC), *n* (%)	7 (5.7)	7 (15.2)
Metastatic adenocarcinoma (rectal origin), n (%)	2 (1.6)	0 (0)

Abbreviations: BTAC, biliary tract adenocarcinoma; PDAC, pancreatic ductal adenocarcinoma; PNEC, pancreatic neuroendocrine carcinoma.

* Excluded one sample due to the patient's request for discontinuation.

**TABLE 2:** Endoscopic ultrasound‐guided tissue acquisition procedure.

	Total (*n* = 123)	F1CDx cases (*n* = 45)	PDAC (*n* = 37)	PNEC (*n* = 1)	BTAC (*n* = 7)
Tumor size (mm)					
≤30	80	27	20		7
>30	43	18	17	1	
22‐gauge needle type					
Acquire	73	36	30		6
Trident Needle Biopsy System	18	5	3	1	1
SonoTip TopGain	14	2	2		
SharkCore	2	1	1		
EZ Shot 3 Plus	15	1	1		
Others	1				
Number of punctures					
1 or 2	6	26	21	1	4
3	64	10	8		2
≥4	35	9	8		1
Target organ					
Pancreatic head/body/tail	42/36/30	10/14/11	10/14/10	0/0/1	0/0/0
Lymph node	8	5			5
Liver	1	1	1		
Biliary duct	1	1			1
Others	5	3	2		1

Abbreviations: BTAC, biliary tract adenocarcinoma; PDAC, pancreatic ductal adenocarcinoma; PNEC, pancreatic neuroendocrine carcinoma.

**TABLE 3:** Tissue area of hematoxylin and eosin‐stained slide prepared by the second sectioning of tissue (F1‐HE) specimens and calculated sample volume.

Tissue area (mm^2^) (*n* = 45)	
Mean, median	18.3, 16.7
<25, *n* (%)	37 (82.2)
≥25, *n* (%)	8 (17.8)
Calculated sample volume (mm^3^) (*n* = 45)	
Mean, median	3.0, 2.8
<1, *n* (%)	1 (2.2)
≥1, *n* (%)	44 (97.8)

Sample volume was calculated using F1‐HE, section thickness, and the number of USSs.

Sample volume, which was calculated on the basis of F1‐HE and the number of USS, Of 45 samples, 43 (95.6%) completed the F1CDx evaluation; results are summarized in Table 4. FMI pathologists microscopically estimated the percentage of tumors, which ranged from 20% to 50% (median, 20%). The success rate was 97.3% (36/37) for PDAC. For BTAC, six of seven (85.7%) tests were successful. In one BTAC sample, although the sample passed the FMI pathologist review for the percentage of nucleated tumor cells, the assay failed after sequencing because computational tumor purity was below the criteria, which reduced sensitivity. Of 43 samples that completed F1CDx testing, nine (20.9%) were “qualified,” which meant that MSS, TMB, or both cannot be determined. The median number of pathological gene mutations examined in the expert panel was 5 (range, 1–11). Of 37 PDAC samples, the numbers of samples with mutations in four major genes (*KRAS*, *TP53*, *CDKN2A*, and *SMAD4*) were 35 (97.2%), 21 (58.3%), 23 (63.9%), and 13 (36.1%), respectively (Table 5). All of the patients with PDAC had at least one variant of these genes. In addition, nine patients (16.7%) had homologous recombination disease‐related genetic mutations, which included *BRCA2* (three patients, 8.3%), *ATM* (three patients, 8.3%), *RAD51C* (two patients, 5.6%), and *PALB2* (one patient, 2.8%). In the six patients with biliary tract cancer, the following mutations were detected: *KRAS* in one patient (16.7%), *TP53* in five (83.3%), *CDKN2A* in four (66.7%), *SMAD4* in three (50.0%), and *CDKN2B* in two (33.3%; Table 6). *TERT* promoter mutations were detected in three patients (50%). Among the above, six genes were identified as candidate druggable abnormalities by the expert panel and four of them were actually treated and listed in Table 6. One patient with PDAC was diagnosed with TMB‐High and treated with pembrolizumab. One with a *BRCA* mutation was treated with olaparib after the BRACAnalysis CDx test (Myriad Genetics, Inc.). Each case with KRAS G12C mutation and PNEC with the *BRAF* V600‐K601>E mutation was led to treatment with either sotorasib or dabrafenib/trametinib, respectively (Table 7).

**TABLE 4:** Tumor cell contents and success rate of sequencing analysis for FoundationOne CDx cancer genome profiling test.

Tumor cell percentages, median (range)	20 (20–50)
20, % (*n*/total *n*)	60.4 (26/43^*^)
25, % (*n*/total *n*)	2.3 (1/43^*^)
30, % (*n*/total *n*)	30.2 (13/43^*^)
35, % (*n*/total *n*)	4.7 (2/43^*^)
40, % (*n*/total *n*)	0.0 (0/43^*^)
50, % (*n*/total *n*)	2.3 (1/43^*^)
Success (pass or qualified), % (*n*/total *n*)	95.6 (43/45)
Histological type	
PDAC, % (*n*/total *n*)	97.3 (36/37)
PNEC, % (*n*/total *n*)	100 (1/1)
BTAC, % (*n*/total *n*)	85.7 (6/7)^**^

Abbreviations: BTAC, Biliary tract adenocarcinoma; PDAC, pancreatic ductal adenocarcinoma; PNEC, pancreatic neuroendocrine carcinoma.

* The tumor proportion data of the F1CDx report for the two cases in which the test was discontinued is not available.

** One re‐biopsy sample included.

**TABLE 5:** Results of Foundation One CDx cancer genome profiling test for pancreatic ductal adenocarcinoma.

Biomarker	*n* (%)
Tumor mutation burden ≥10	1 (2.8)
*KRAS*	35 (97.2)
*CDKN2A*	23 (63.9)
*TP53*	21 (58.3)
*CDKN2B*	17 (47.2)
*MTAP*	14 (38.9)
*SMAD4*	13 (36.1)
*ARID1A*	6 (16.7)
*ATM*	3 (8.3)
*BRCA2*	3 (8.3)
*MYC*	3 (8.3)
*FGFR3*	2 (5.6)
*MAP*	2 (5.6)
*RAD51C*	2 (5.6)
*STK11*	2 (5.6)
*U2AF1*	2 (5.6)
*AKT3*	1 (2.8)
*BRAF*	1 (2.8)
*ERBB2*	1 (2.8)
*FGFR4*	1 (2.8)
*MET*	1 (2.8)
*PIK3CA*	1 (2.8)
*TERC*	1 (2.8)
*PIK3CA*	1 (2.8)
*TERC*	1 (2.8)

° Four major representative driver mutations.

**TABLE 6:** Results of FoundationOne CDx cancer genome profiling test for biliary tract adenocarcinoma.

Biomarker	*n* (%)
Tumor mutation burden ≥10	0 (0.0)
*TP53*	5 (83.3)
*CDKN2A*	4 (66.7)
*SMAD4*	3 (50.0)
*TERT* promoter ‐124C > T	3 (50.0)
*CDKN2B*	2 (33.3)
*MAP*	2 (33.3)
*MTAP*	2 (33.3)
*ARID1A*	1 (16.7)
*BRAF*	1 (16.7)
*KRAS*	1 (16.7)
*STK11*	1 (16.7)

**TABLE 7:** A list of druggable gene abnormalities and the therapeutic drugs utilized.

Actionable alteration	*n*	Histological type	Candidate drug	Therapeutic drugs
BRAF V600_K601 > E	1	PNEC	BRAF inhibitor + MEK inhibitor	Dabrafenib‐Trametinib
BRCA2	1	PDAC	PARP inhibitor	Olaparib
KRAS G12C	1	PDAC	KRAS G12C inhibitor	Sotorasib
Tumor mutation burden				
10 Mut/Mb	1	PDAC	Immune checkpoint inhibitor	Pembrolizumab

Abbreviations: PDAC, pancreatic ductal adenocarcinoma; PNEC, pancreatic neuroendocrine carcinoma.

## DISCUSSION

In this study, we reported results from a single institution where F1CDx testing using EUS‐TA specimens obtained with 22G needles achieved a 90.8% evaluation rate based on a pathologist review, with 43 of 45 samples (95.6%) successfully tested. Our results demonstrate that tissue samples obtained with 22G needles are sufficiently suitable for clinical F1CDx testing. Such comprehensive reports have not been previously reported (Table 7). In Japan, F1CDx and NCCOP have been covered by health insurance since June 2019 and are primarily used for CGP. According to Yamai et al., F1CDx is often preferred in pancreatic cancer with its multiple companion functions, with 93 of 115 specimens (80.9%) tested with F1CDx and 22 (19.1%) tested with NCCOP.^21^ Some studies on CGP with EUS‐TA samples have much higher ratios of NCCOP to F1CDx, likely due to NCCOP requiring less tissue volume.^15^, ^16^, ^18^ Data on the use of 22G samples in F1CDx were very limited; some reports did not specify the success rate. While needle type, size, number of punctures, tissue fragment length, and presence of MOSE have been studied in relation to CGP using EUS‐TA samples, inadequate evaluations by pathologists often result in low success rates.^14^, ^21^, ^22^ A previous report showed that samples obtained with 22G needles have a low rate of meeting the 25 mm^2^ standard required by F1CDx.^13^ Since 2019, we have aimed not to adopt the 25 mm^2^ standards in the pathologist adequacy check, but instead to produce enough USS to meet the alternative standard of 1 mm^3^ based on needle gauge and tissue length, which we call the SMAGLE method (Figure 3). Given a 20 mm white MVC in a 22G needle, producing 25 USS would theoretically meet the required sample volume. As shown in Table 3, sample volume exceeded 1 mm^3^ in all except one specimen. In fact, FMI pathologists evaluate the surface area of the submitted HE specimen (F1‐HE) and determine the number of USS needed. If it does not meet the 25 mm^2^ standard, they use additional USS according to their 1 mm^3^ criteria. Our adequacy evaluation and slide preparation SMAGLE methods can be in line with F1CDx testing procedures. A 20 mm long MVC in a 22G needle has a volume of approximately 2.64 mm^3^ (volume of cylindrical tissue 0.41 mm in diameter and 20 mm in length); 1 mm^3^ corresponds to approximately 37.9% of this volume (Figure 3). Therefore, the sectioning using the TiPS method instead of the conventional approach should be carried out (Figure 2). Significant differences in the area among Dx‐HE, F1‐HE, and End‐HE in our study suggest the usefulness of the TiPS in small samples. Additionally, no problems were found with pathological diagnosis using Dx‐HE made by the TiPS.

Finally, the low adequacy evaluation has significantly reduced the success rate of F1CDx with tissue obtained via EUS‐TA (Table 8).^11^, ^12^, ^13^, ^14^, ^15^ In tissue samples acquired with a 22G needle, the 25 mm^2^ requirement should not be a firm rule. We clarified that F1CDx is possible with TiPS and SMAGLE by increasing the number of USS as Ikeda et al. previously speculated and that both pathological diagnosis and CGP can be performed with a single FFPE tissue.^13^

**TABLE 8:** Success rate of Foundation One CDx (F1CDx) and the OncoGuide NCC Oncopanel system (NCCOP) using formalin‐fixed paraffin‐embedded tissues from endoscopic ultrasound‐guided fine needle acquisition (EUS‐TA): a literature review.

First author (year of publication) Study design	EUS‐TA Total: *N* Histological type (*n*)	Needle size and type	Type of CGP	Histological adequacy (Pre‐check), % (*n*/total *n*)	CGP success rate of sequencing analysis in CGP, % (*n*/total *n*)	Mutation rates in four major PDAC genes, %
Larson et al. (2018) Retrospective	61 PDAC (61)	19‐25G EUS‐FNA 19–25G EUS‐FNB Other	F1CDx	67.2% (41/61)	N/A	N/A
Kandel (2021) Prospective	50 PDAC (37) PNET (5) Other (8)	19G or 22G EUS‐FNB	F1CDx	78% (39/50)	N/A	N/A
Ikeda et al. (2022) Retrospective	153 PDAC (153)	19G EUS‐FNB 22G EUS‐FNA 22G EUS‐FNB	F1CDx NCCOP	F1CDx 0% (0/153) NCCOP 39.2% (60/153)	Total 100% (30/30) 22G N/A F1CDx 22G 0% (0/0)	*KRAS* 93.3% *TP53* 76.7% *SMAD4* 30.0% *CDKN2A* 20.0%
Hisada et al. (2022) Prospective	33 PDAC (31) Other (2)	19G EUS‐FNB	F1CDx NCCOP	F1CDx 0% (0/33) NCCOP 63.6% (21/33)	Total 100% (12/12) 22G 0% (0/0) F1CDx 22G 0% (0/0)	*KRAS* 100% *TP53* 66.7% *SMAD4* 66.7% *CDKN2A* 16.6%
Okuno et al. (2023) Retrospective	109 PDAC (129)^*^ PNET (11)^*^ Other (11)^*^	19G EUS‐FNB 22G EUS‐FNA 22G EUS‐FNB	F1CDx NCCOP	57.8% (63/109)	Total 96.8% (61/63) 22G N/A F1CDx 22G N/A	*KRAS* 92.7%^*^ *TP53* 72.2%^*^ *SMAD4* 34.0%^*^ *CDKN2A* 29.9%^*^
Yanaidani et al. (2024) Retrospective	137 BTAC (137)	19G EUS‐FNB 22G EUS‐FNB Others	F1CDx NCCOP Other	82.4% (113/137)^*^	Total 76.5% (39/51) 22G 60.0% (12/20) F1CDx 22G N/A	N/A
Ishigaki et al. (2024) Retrospective	50 PDAC (39) BTAC (8) Other (3)	22G EUS‐TA	F1CDx NCCOP	86.0% (43/50)	Total 93.0% (38/41) 22G 93.0% (38/41) F1CDx 22G 100% (2/2)	N/A
Present study Retrospective	119 PDAC (109) PNEC (1) BTAC (9)	22G EUS‐TA	F1CDx	F1CDx 90.8% (108/119)	Total 95.6% (43/45) 22G 95.6% (43/45) F1CDx 22G 95.6% (43/45)	*KRAS* 97.2% *TP53* 58.3 % *SMAD4* 36.1 *CDKN2A* 63.9%

Abbreviations: BTAC, biliary tract adenocarcinoma; CGP, comprehensive genome profiling; EUS‐FNA, endoscopic ultrasound‐guided fine needle aspiration; EUS‐FNB, endoscopic ultrasound‐guided fine needle biopsy; EUS‐TA, endoscopic ultrasonography‐guided tissue acquisition; F1CDx, Foundation OneCDx cancer genome profiling; N/A, not assessable; NCCOP, OncoGuide NCC Oncopanel system; PDAC, pancreatic ductal adenocarcinoma; PNET, pancreatic neuroendocrine tumor; PNEC, pancreatic neuroendocrine carcinoma.

* Surgical specimens and percutaneous biopsy specimens included.

## CONCLUSION

This study clarified a high adequacy evaluation (90.8%) and high success rate (95.6%) for F1CDx testing using 22G EUS‐TA samples, demonstrating the clinical suitability of their use. Puncturing 2–3 times followed by confirming white tissue fragments of 20 mm or more is crucial. Reducing FFPE tissue loss and submitting 30 or more USS to provide a sample volume of 1 mm^3^ lead to a high F1CDx success rate.

## CONFLICT OF INTEREST STATEMENT

None.

## ETHICS STATEMENT

This study was conducted in accordance with the Declaration of Helsinki. Ethics approval was obtained from the ethics review committee of the Himeji Red Cross Hospital (Approval No. 2023–64).

## PATIENT CONSENT STATEMENT

As this was a retrospective study, the requirement for informed consent was waived, and an opt‐out method was provided on our hospital's website.

## CLINICAL TRIAL REGISTRATION

N/A.

## References

[1] Banafea O , Mghanga FP , Zhao J *et al*. Endoscopic ultrasonography with fine‐needle aspiration for histological diagnosis of solid pancreatic masses: A meta‐analysis of diagnostic accuracy studies. BMC Gastroenterol 2016; 16: 108. 27580856 10.1186/s12876-016-0519-z PMC5007683

[2] Takano Y , Noda J , Yamawaki M *et al*. Comparative study of an ultrasound‐guided percutaneous biopsy and endoscopic ultrasound‐guided fine‐needle aspiration for liver tumors. Intern Med 2021; 60: 1657–1664. 34078770 10.2169/internalmedicine.6183-20 PMC8222129

[3] Japan Pancreas Society . NCCN Guidelines for Pancreatic Cancer. [Internet]. [cited 2024 Aug 13]. Available from: https://www2.tri‐kobe.org/nccn/guideline/pancreas/japanese/pancreatic.pdf (In Japanese).

[4] Benson AB , D'Angelica MI , Abrams T *et al*. NCCN guidelines insights: Biliary tract cancers, version 2.2023. J Natl Compr Canc Netw 2023; 21: 694–704. 37433432 10.6004/jnccn.2023.0035

[5] Foundation Medicine Inc . FoundationOne CDx: Technical Information [Internet]. [no date; cited 2024 Jul 26]. Available from: https://info.foundationmedicine.com/hubfs/FMI%20Labels/FoundationOne_CDx_Label_Technical_Info.pdf

[6] Foundation Medicine Inc ., FoundationOne CDx: Specimen Instructions [Internet], Cambridge, MA: Foundation Medicine Inc., 2024. Available from: https://www.foundationmedicine.com/sites/default/files/media/documents/2024‐04/F1CDx_Specimen_Instructions.pdf

[7] OncoGuide NCC oncopanel system: Specimen instructions (in Japanese.) [Internet]. Available from: OncoGuide_Sample‐preparation‐guide.pdf (sysmex.co.jp)

[8] Elhanafi S , Mahmud N , Vergara N *et al*. Comparison of endoscopic ultrasound tissue acquisition methods for genomic analysis of pancreatic cancer. J Gastroenterol Hepatol 2019; 34: 907–913. 30422342 10.1111/jgh.14540 PMC6497552

[9] Gan Q , Roy‐Chowdhuri S , Duose DY *et al*. Adequacy evaluation and use of pancreatic adenocarcinoma specimens for next‐generation sequencing acquired by endoscopic ultrasound‐guided FNA and FNB. Cancer Cytopathol 2022; 130: 275–283. 34905283 10.1002/cncy.22533

[10] Kandel P , Nassar A , Gomez V *et al*. Comparison of endoscopic ultrasound‐guided fine‐needle biopsy versus fine‐needle aspiration for genomic profiling and DNA yield in pancreatic cancer: A randomized crossover trial. Endoscopy 2021; 53: 376–382. 32767288 10.1055/a-1223-2171

[11] Larson BK , Tuli R , Jamil LH *et al*. Utility of endoscopic ultrasound‐guided biopsy for next‐generation sequencing of pancreatic exocrine malignancies. Pancreas 2018; 47: 990–995. 30028448 10.1097/MPA.0000000000001117

[12] Park JK , Lee JH , Noh DH *et al*. Factors of endoscopic ultrasound‐guided tissue acquisition for successful next‐generation sequencing in pancreatic ductal adenocarcinoma. Gut Liver 2020; 14: 387–394. 31581388 10.5009/gnl19011 PMC7234878

[13] Ikeda G , Hijioka S , Nagashio Y *et al*. Fine‐needle biopsy with 19G needle is effective in combination with endoscopic ultrasound‐guided tissue acquisition for genomic profiling of unresectable pancreatic cancer. Dig Endosc 2023; 35: 124–133. 35993898 10.1111/den.14423

[14] Hisada Y , Hijioka S , Ikeda G *et al*. Proportion of unresectable pancreatic cancer specimens obtained by endoscopic ultrasound‐guided tissue acquisition meeting the OncoGuide NCC Oncopanel system analysis suitability criteria: A single‐arm, phase II clinical trial. J Gastroenterol 2022; 57: 990–998. 36190682 10.1007/s00535-022-01926-z

[15] Okuno N , Hara K , Mizuno N *et al*. Clinical utility of endoscopic ultrasound‐guided tissue acquisition for comprehensive genomic profiling of pancreatic cancer. Clin Endosc 2023; 56: 221–228. 36879539 10.5946/ce.2022.086 PMC10073855

[16] Yanaidani T , Hara K , Okuno N *et al*. Clinical utility of endoscopic ultrasound‐guided tissue acquisition for comprehensive genomic profiling of patients with biliary tract cancer, especially with intrahepatic cholangiocarcinoma. Clin Endosc 2024; 57: 384–392. 38356172 10.5946/ce.2023.139 PMC11133989

[17] Hijioka S , Nagashio Y , Maruki Y *et al*. Endoscopic ultrasound‐guided tissue acquisition of pancreaticobiliary cancer aiming for a comprehensive genome profile. Diagnostics 2023; 13: 1275. 37046493 10.3390/diagnostics13071275 PMC10093621

[18] Ishigaki K , Nakai Y , Endo G *et al*. Feasibility of comprehensive genomic profiling using endoscopic ultrasound‐guided tissue acquisition with a 22‐gauge Franseen needle. DEN Open 2024; 4: e365. 38628502 10.1002/deo2.365 PMC11019146

[19] Iwashita T , Yasuda I , Mukai T *et al*. Macroscopic on‐site quality evaluation of biopsy specimens to improve the diagnostic accuracy during EUS‐guided FNA using a 19‐gauge needle for solid lesions: A single‐center prospective pilot study (MOSE study). Gastrointest Endosc 2015; 81: 177–185. 25440688 10.1016/j.gie.2014.08.040

[20] Kanda Y . Investigation of the freely available easy‐to‐use software ‘EZR’ for medical statistics. Bone Marrow Transplant 2013; 48: 452–458. 23208313 10.1038/bmt.2012.244 PMC3590441

[21] Yamai T , Ikezawa K , Sugimoto N *et al*. Utility of comprehensive genomic profiling tests for patients with incurable pancreatic cancer in clinical practice. Cancers 2023; 15: 970. 36765927 10.3390/cancers15030970 PMC9913675

[22] Woo SM . Endoscopic ultrasound‐guided tissue acquisition for personalized treatment in pancreatic adenocarcinoma. Clin Endosc 2023; 56: 183–184. 36941791 10.5946/ce.2023.037 PMC10073856

